# Beyond Substituted *p*-Phenylenediamine Antioxidants: Prevalence of Their Quinone Derivatives in PM_2.5_

**DOI:** 10.1021/acs.est.2c02463

**Published:** 2022-07-14

**Authors:** Wei Wang, Guodong Cao, Jing Zhang, Pengfei Wu, Yanyan Chen, Zhifeng Chen, Zenghua Qi, Ruijin Li, Chuan Dong, Zongwei Cai

**Affiliations:** † State Key Laboratory of Environmental and Biological Analysis, Department of Chemistry, Hong Kong Baptist University, Hong Kong SAR 999077, China; ‡ School of Environmental Science and Engineering, Guangdong University of Technology, Guangzhou 510006, China; § Institute of Environmental Science, Shanxi University, Taiyuan 030006, China

**Keywords:** tire rubber additives, *para*-phenylenediamine derivatives, fine particulate matter, airborne quinones, human inhalation exposure

## Abstract

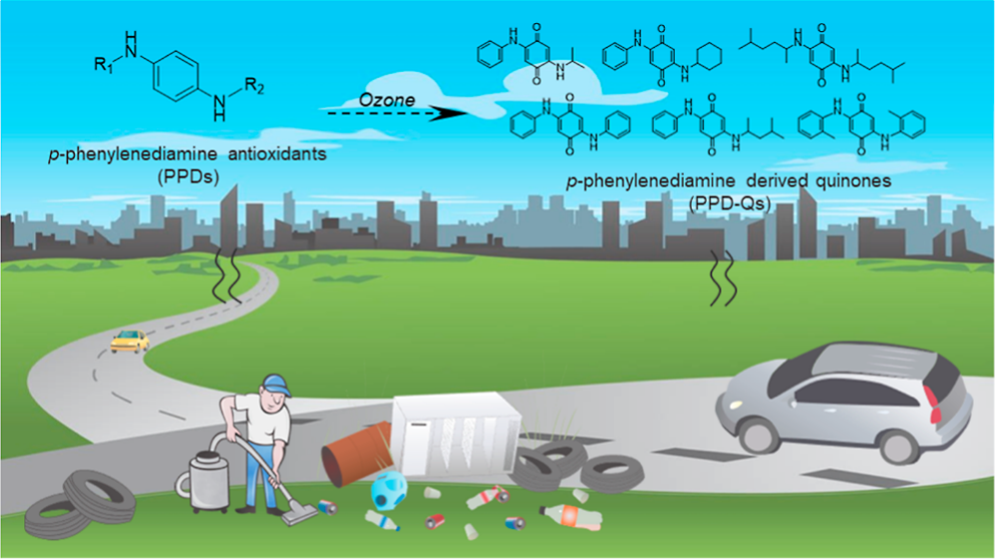

Substituted *para*-phenylenediamine (PPD) antioxidants have been extensively used to retard oxidative degradation of tire rubber and were found to pervade multiple environmental compartments. However, there is a paucity of research on the environmental occurrences of their transformation products. In this study, we revealed the co-occurrence of six PPD-derived quinones (PPD-Qs) along with eight PPDs in fine particulate matter (PM_2.5_) from two Chinese megacities, in which *N*,*N*′-bis(1,4-dimethylpentyl)-*p*-phenylenediamine quinone (77PD-Q) was identified and quantified for the first time. Prevalent occurrences of these emerging PPD-Qs were found in Taiyuan (5.59–8480 pg/m^3^) and Guangzhou (3.61–4490 pg/m^3^). Significantly higher levels of PPDs/PPD-Qs were observed at a roadside site, implying the possible contribution of vehicle emissions. Correlation analysis implied potential consistencies in the fate of these PPD-Qs and suggested that most of them were originated from the transformation of their parent PPDs. For different subpopulation groups under different exposure scenarios, the estimated daily intakes of PPD-Qs (0.16–1.25 ng kg_bw_^–1^ day^–1^) were comparable to those of their parent PPDs (0.19–1.41 ng kg_bw_^–1^ day^–1^), suggesting an important but overlooked exposure caused by novel PPD-Qs. Given the prolonged exposure of these antioxidants and their quinone derivatives to traffic-relevant occupations, further investigations on their toxicological and epidemiological effects are necessary.

## Abstract 2

This study revealed the co-occurrence of six substituted *para*-phenylenediamine-derived quinones with their parent compounds in the PM_2.5_ from two typical megacities in China and comprehensively evaluated their inhalation exposure.

## Introduction

Substituted *para*-phenylenediamines (PPDs), as a class of anthropogenic antioxidants, have been largely produced and applied in the rubber industry due to their capability for the protection of rubber materials against flex cracking, heat degradation, and ozone cracking.^1^ However, massive production and consumption of these PPD antioxidants have caused increasing concern regarding their emissions and potential influence on the aquatic and terrestrial ecosystems. Emerged evidence indicated that these chemicals can be ubiquitously detected in receiving waters,^2,3^ dusts,^4,5^ sediments,^6^ and air particles.^7^ In parallel, detrimental effects of several broadly adopted PPDs have been reported. N, *N*′-diphenyl-*p*-phenylenediamine (DPPD) was found to cause reproductive/developmental toxicity in rats.^8^*N*-1, 3-dimethylbutyl-n′-phenyl-*p*-phenylenediamine (6PPD) was proved to be toxic to aquatic organisms, with the 48 h larvae viability inhibition half-maximal effective concentration (EC_50_) for two freshwater mussels ranging from 137 to 439 mg/L, while its total survival 21-d median lethal concentration (LC_50_) for the early life stages of the fathead minnow was 35 mg/L.^9,10^ In addition, *N*-isopropyl-m′-phenyl-*p*-phenylenediamine (IPPD) was defined as predominantly occupational allergens, causing allergic contact dermatitis through direct contact with industrial rubber.^11^ A test report from the U.S. EPA has illustrated the acute toxicity of *N*,*N*′-bis(1,4-dimethylpentyl)-*p*-phenylenediamine (77PD) to rats through inhalation with a LC_50_ of 400 mg/m^3^.^12^

In addition to PPDs, the environmental emissions and acute toxicity of their derived quinones (PPD-Qs) have attracted great attention lately. In a recent study, *N*-(1,3-dimethylbutyl)-*N*′-phenyl-*p*-phenylenediamine quinone (6PPD-Q), a transformation product of 6PPD, was identified in roadway runoff and runoff-affected receiving waters, which has been demonstrated to be highly toxic and can cause acute mortality (24 h LC_50_ of 95 ng/L) of coho salmon (*Oncorhynchus kisutch*) in the Pacific Northwest before they spawn in freshwater streams.^13,14^ Apart from the coho salmon, 6PPD-Q was also proved to be toxic to zebrafish larvae with a 24 h LC_50_ of 308.67 μg/L.^15^ Newer data indicated that this emerging contaminant was pervasive in urban road runoff, watersheds, and indoor/outdoor dust.^3,4^ More recently, our study first demonstrated the widespread distribution of 6PPD-Q and other multiple PPDs/PPD-Qs in urban runoff and roadside soils and also revealed their occurrence in fine particulate matter (specifically, particulate matter smaller than 2.5 μm, referred to as PM_2.5_) samples collected in Hong Kong, China.^16^

As a complex matrix that is highly influenced by the surroundings, PM_2.5_ can absorb a variety of organic/inorganic substances due to its high specific surface area.^17^ Appreciable efforts have been made to investigate the occurrences of polyaromatic hydrocarbons (PAHs), metals, water-soluble ions, and elemental/organic carbon in fine particles,^18,19^ demonstrating a wide range of health effects, for instance, causing acute oxidative stress, inflammation, DNA damage, and pulmonary impairment.^20−22^ In China, it was reported that as much as 40.3% of total stroke deaths, 23.9% of lung cancer deaths, and 15.5% of all-cause deaths were related to the exposure to PM_2.5_ in the year 2015 alone.^23^ However, there is still a striking discrepancy between the high concentrations of these chemicals tested in toxicologic studies and the low concentrations of their presence analyzed in real environmental PM_2.5_. Thus, analysis of emerging contaminants in PM_2.5_ samples and consequent evaluation of their potential health effects are of great significance. Given the large appliances of various PPDs (∼100,000 tons consumption in 2009) and severe air pollution in China, the inquiry into screening PPD-Qs in the PM_2.5_ samples is far from sufficient.^24^ Their concentration and composition profiles could probably be significantly varied, and other unrevealed PPD-Qs may also be present in PM_2.5_. In this study, we aim to (1) identify and quantify PPDs/PPD-Qs in PM_2.5_ samples, (2) compare their levels and spatiotemporal variations from different sites in China, (3) investigate the associations between PPD-Qs and PPDs, and (4) estimate the exposure levels of humans to PM_2.5_-bound PPD-Qs and PPDs via inhalation.

## Materials and Methods

### Standards and Reagents

The measured compounds in this study included eight substituted PPDs and six quinone derivatives (PPD-Qs). Their specific name, abbreviation, Chemical Abstracts Registry Number (CAS No.), and structures are shown in Table S1 and Figure S1. Authentic standards of PPDs were purchased from J & K Chemical Company (Hong Kong), AccuStandard (Hong Kong), and TCI (Hong Kong), while PPD-Q standards were synthesized in our laboratory. Surrogate standard diphenylamine-d_10_ was purchased from TRC (Burlington, Canada). The internal standard of deuterated *N*-(1,3-dimethylbutyl)-*N*′-phenyl-*p*-phenylenediamine quinone (6PPD-Q-d_5_) was synthesized in the laboratory. All the purchased standards were more than 97% in their purity, while the purities of the synthesized standards were estimated to be 95 to 98% based on the total ^1^H NMR integral.^16^ The ^1^H NMR and ^13^C NMR spectra of the quinone of 77PD are illustrated in Figure S2. All the solvents used in this study were of HPLC grade or higher.

### Sample Preparation and Instrument Analysis

Sampling campaigns were conducted at three sites in China located at Shanxi University in Taiyuan (TY, *N* = 24), Guangdong University of Technology in Guangzhou (GZ, *N* = 24), and a roadside sampling site near South China Institute of Environmental Protection in Guangzhou (RS, *N* = 24). Detailed sampling dates and geographical characteristics of the sampling sites are given in Table S2 and Figure S3. The specific geographical locations and collection approaches have been described in our earlier work.^25^ Generally, a 24 h PM_2.5_ sample (∼126 m^3^) was collected on the quartz fiber filter (QMA, 90 mm, Whatman International Ltd, UK) through a medium-volume air sampler (AMAE Co. Ltd, Shenzhen, China) from May 2017 to April 2018. The quartz filter was pre-baked for 5 h at 550 °C in advance to eliminate possible contaminants and stored in a −80 °C freezer wrapped in aluminum foil before further analysis. A whole filter was cut and placed into a 15 mL glass tube, spiked with surrogate standards for 20 ng, ultrasonicated twice for 15 min with 5 mL of dichloromethane, and then the ultrasonic extraction was repeated for another 15 min with 5 mL of acetonitrile. The extract was combined and concentrated to near dryness by nitrogen purge. After solvent exchange with 1 mL of acetonitrile, the extract was filtered with a 0.45 μm PTFE organic filter membrane and spiked with 20 ng of internal standard before instrument analysis.

Instrument analysis was performed by a combination of electrospray ionization (ESI), ultrahigh-resolution Orbitrap mass spectrometry (MS), and triple quadrupole MS. A Q Exactive hybrid quadrupole-Orbitrap mass spectrometer (Thermo Scientific, USA) was used to identify these analytes in the data-dependent MS^2^ mode, while their quantification was conducted with a TSQ Altis MS system (Thermo Scientific, USA) in the multiple reaction monitoring mode. A Thermo Vanquish MD HPLC system was used for separation, and the particular chromatographic conditions, qualify/quantify ion pairs, and analyte-dependent operational parameters are listed in Tables S3 and S4.^16^

### Quality Control, Quality Assurance, and Data Analysis

To evaluate possible contaminations caused by the sampling and pretreatment procedures, field blank samples consisting of quartz fiber filters were transported, stored, and extracted in the same manner as atmospheric particle samples. Analytes including CPPD, 6PPD, DPPD, DTPD, IPPD-Q, and CPPD-Q were detectable in the blank samples with abundance of <2% of their quantified levels and were subtracted as background levels. Six replicates of a pre-baked quartz filter and real PM_2.5_ absorbed filters spiked with 10 ng of target analytes were used to assess the blank recoveries and matrix recoveries of analytical procedures. The matrix spike recoveries of these target analytes obtained from the spiking analysis ranged from 70 ± 2 to 97 ± 4% (Table S4). As the recoveries of the analytes were all in the range of 70–130%, the normalization of the concentration was not conducted to their observed recoveries. The repeatability of the method was assessed by a duplicate test for every eight samples, and the standard deviations were all less than 20%. The calibration curve for each analyte was made in the acetonitrile solvent, with all their regression coefficients being higher than 0.99. Samples were diluted if their concentrations were beyond the range of the calibration curve. The calculation of method detection limits (MDLs) and method quantification limits (MQLs) for different analytes varied according to their occurrence in the blank samples and their recoveries (Table S4). For analytes that were detected in the blank, the MDLs/MQLs were defined as 3/10 times the standard deviation of the procedural blank; while for analytes that were not detectable in blank samples, the MDLs/MQLs were calculated using 3/10 times S/N ratios of the lowest detectable levels for the standards dissolved in the matrix.

The processing of the LC-MS spectrum was applied with Xcalibur software (V4.3.7, Thermo Scientific, USA). Non-parametric statistical analysis methods like the Mann–Whitney test, Spearman correlations, and others were performed using SPSS 11.0 (IBM, SPSS Inc.). A *p*-value less than 0.05 was considered statistically significant. Detailed calculations of human exposure under different scenarios are presented in the Supporting Information.

## Results and Discussion

### Identification of a Novel PPD-Derived Quinone in PM_2.5_ Samples

**Figure 1:**
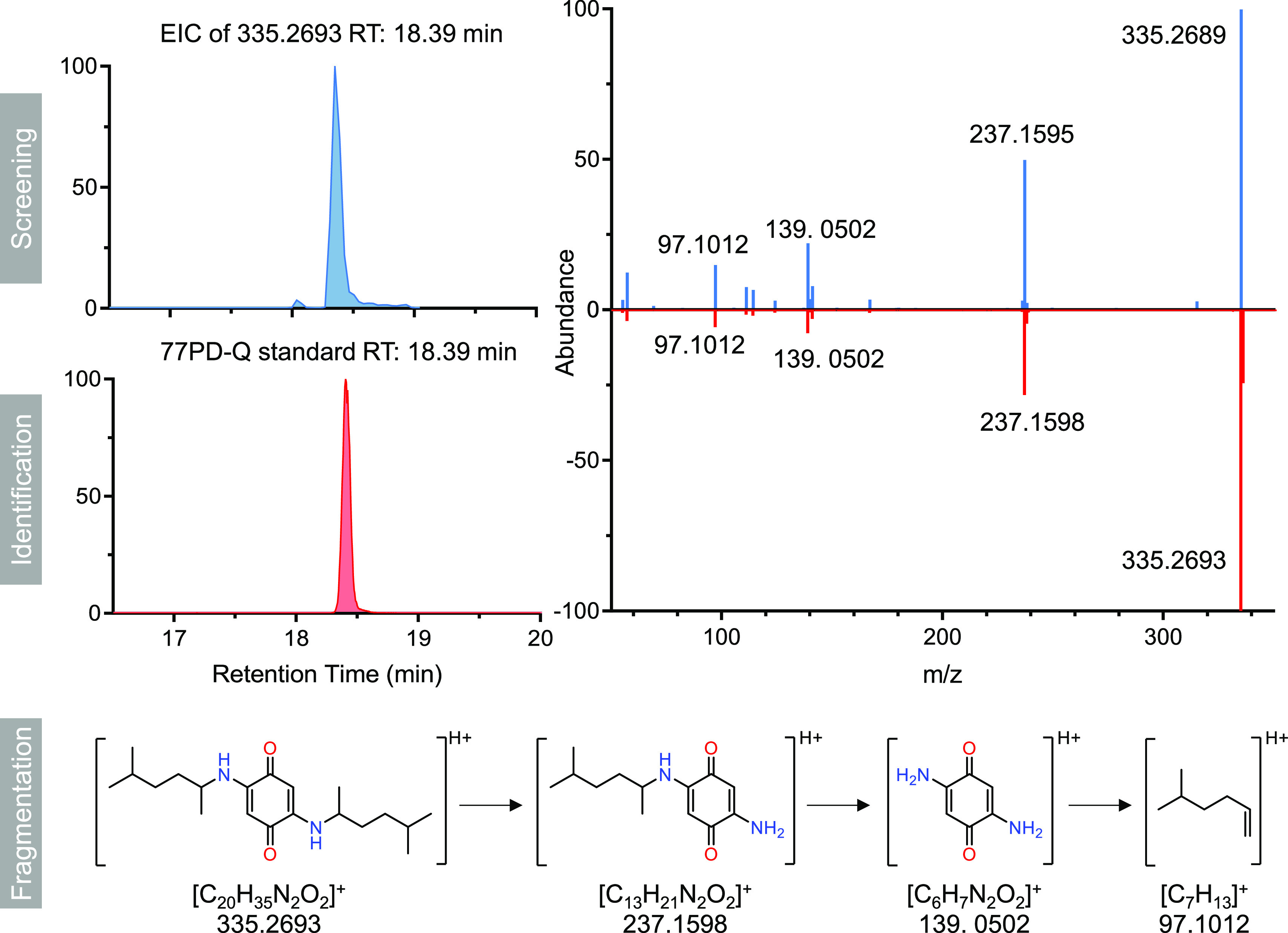
Extracted ion chromatogram of the [M + H]^+^ ion of 77PD-Q and its MS^2^ fragments from the real PM_2.5_ sample and the synthesized 77PD-Q standard and the potential structures of the fragments of 77PD-Q.

Previous studies have reported various levels of PPDs among multiple environmental matrices and scenarios.^5,6^ Huang et al. have investigated the occurrence of PPDs in the dust among different intracity scenarios including roads, parking lots, vehicles, and houses, suggesting a diverse concentration and composition profiles of these chemicals.^4^ In another study, Zhang et al. evaluated the level of PPDs in PM_2.5_ among six different cities in China and found remarkable intercity distinctions.^7^ Their results found that the levels of 77PD were comparable or even higher than 6PPD in the investigated six cities. Considering this, we speculated that 77PD-Q may also occur in PM_2.5_ collected from highly polluted areas/cities. Therefore, we utilized high-resolution MS for target screening of 77PD-Q in PM_2.5_ obtained from Taiyuan and Guangzhou, China, which were reported to suffer from serious atmospheric pollution during 2017.^26,27^ First, we have searched for the exact mass of the [M + H]^+^ ion (*m/z* 335.2693) in the PM_2.5_ samples, where a strong base peak at *m/z* 335.2689 with a retention time (RT) of 18.39 min was observed (Figure 1). The differentiation between the measured mass and theoretical mass was only −1.19 ppm, implying a high probability of the occurrence of 77PD-Q in the PM_2.5_ samples. To further clarify the identity of this chromatography peak, we have synthesized the standard of 77PD-Q via Michael-type addition of 2-amino-5-methylhexane and benzoquinone (Supporting Information). As shown in Figure 1, a perfect match in both MS^2^ fragments and RT in chromatography was found between the standard substance and the suspect peak detected in PM_2.5_ samples. Among them, fragment ions at *m/z* 237.1595 and 139.0502 can be attributed to the sequential losses of C_7_H_15_ from both sides of 77PD-Q. Meanwhile, another fragment ion at *m/z* 97.1012 indicated the broken branch chain of C_7_H_13_^+^. These results collectively indicated that 77PD-Q was present in the PM_2.5_ samples, and to the best of our knowledge, this is the first report of its occurrence.

Besides this, we have also observed other PPD-Qs and PPDs in the PM_2.5_ samples. The results indicated that a total of six PPD-Qs (Figure S4) along with their parent compounds were detectable in the extracts. The identification of these quinones was confirmed from their exact mass, MS/MS spectra, and RT match to the self-synthetic standard. It is worth noting that these quinones exhibit distinct MS/MS fragmentation pathways (Figure S5). Among them, DPPD-Q and DTPD-Q bearing symmetrical arylamines are prone to cleavage at the C=O double bond, yielding ions at *m*/*z* 263.1179 and 291.1492, respectively. Otherwise, asymmetric chain-substituted diphenylamine quinones like IPPD-Q, CPPD-Q, and 6PPD-Q are likely to show fragmentation from their side chains, generating a common ion peak at *m*/*z* 215.0815, which is rationalized by the loss of C_3_H_6_, C_6_H_10_, and C_6_H_12_, respectively. Next, we have performed HPLC-ESI triple quadrupole MS to achieve the quantification of these emerging contaminants by taking these observations into account for the construction of precursor–product ion pairs.

### Concentrations and Composition Profiles of PPDs and PPD-Qs in PM_2.5_ Samples

**Figure 2:**
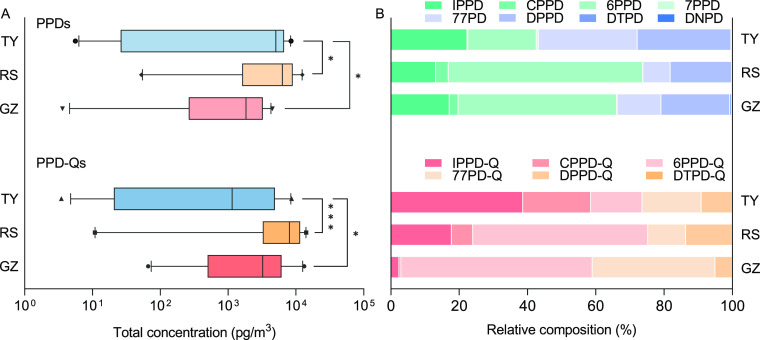
Total concentrations (A) of PPDs and PPD-Qs and their composition profiles (B) at site Guangzhou (GZ), roadside of Guangzhou (RS), and Taiyuan (TY).

Table 1 illustrates the quantitative results of PPD-Qs and PPDs in PM_2.5_ samples from Guangzhou and Taiyuan, two megacities each in South and North China with significantly different geographical and economic structures. It can be seen that except for DTPD-Q, all the other PPD-Qs, along with their parent compounds, showed a high detection frequency (DF) of more than a half, indicating a prevalent occurrence of these antioxidants and their quinone derivatives bonded to the atmospheric particulates. The total concentrations of these measured PPD-Qs at the Guangzhou site varied from 3.61 to 4490 pg/m^3^ (median of 1830 pg/m^3^), which is significantly lower (*p* < 0.05) than those at the Taiyuan site (range 5.59–8480 pg/m^3^ and median of 5040 pg/m^3^). Among these, 6PPD-Q is particularly abundant at the Guangzhou site (median of 1100 pg/m^3^), which comprises more than 50% of the total PPD-Qs, while that in Taiyuan only accounts for 15%, with a median level of 744 pg/m^3^ (Figure 2B). The same trend was also observed in its parent compound, 6PPD, which showed the highest proportion among these antioxidants in Guangzhou (46%), with a median level of 1820 pg/m^3^, while that in Taiyuan was only 20%, with a median value of 81.0 pg/m^3^ (Table 1). By contrast, CPPD-Q and IPPD-Q were the dominant species for PPD-Qs in Taiyuan, which accounted for about 20 and 39% of the total of PPD-Qs, respectively. Apart from 6PPD-Q, 77PD-Q also showed a relatively high abundance in Guangzhou (range 0.57–2990 pg/m^3^ and median of 527 pg/m^3^), and its proportion was significantly higher than that in Taiyuan. Such a finding was consistent with the measured concentrations of its parent compound 77PD, which showed distinct abundance variations between Guangzhou (median of 413 pg/m^3^) and Taiyuan (median of 3.78 pg/m^3^). Among the suites of PPD-Qs, DTPD-Q was found to exhibit the lowest environmental concentrations and DF in the two megacities. This observation can be rationalized by the relatively low abundance of DTPD in the PM_2.5_ samples (Table 1). Intriguingly, we noticed that as an analogue of DTPD-Q, DPPD-Q exhibited different abundance patterns in Guangzhou (median of 41.5 pg/m^3^) and Taiyuan (median of 552 pg/m^3^), particularly when concentrations were compared to its parent compound. Its levels in Guangzhou are much lower than DPPD, whereas it is clearly greater in Taiyuan. These results provided parallel evidence indicating the disparate concentrations and compositions of PPD-Qs and PPDs in Taiyuan and Guangzhou in North and South China. The two megacities have significantly different geographical characteristics and economic structures, of which Taiyuan is a typical valley basin city driven by mining and heavy industries, whereas Guangzhou is a port city with light industries and manufacturing, but heavy traffic. Former studies indicated that Taiyuan is favorable for the accumulation of PM_2.5_ and its bounded chemical species, including PAHs, due to its relatively closed basin topography and temperate monsoon climate.^28^ Comparatively, the topography of Guangzhou is relatively flat, making it favorable for the dispersion and transport of PM_2.5_.^29^ There is evidence that vehicle-related or, more specifically, rubber tire-related sources may affect the concentrations and composition profiles of PPDs in various environments.^4^ The number of traffic passengers and motor vehicles in Guangzhou was 7.15 billion and 2.34 million in 2017, while the number in Taiyuan was 0.90 billion and 1.45 million, which might contribute to the dissimilarities in PPD-Qs and PPDs between the two megacities.^30,31^ The similar trend has been reported in the measurement of benzothiazole and its derivatives (BTHs), another kind of rubber antioxidant, in the PM_2.5_ of Guangzhou (median of 564 pg/m^3^) compared to Taiyuan (305 pg/m^3^).^32^ Therefore, we preliminarily conclude that variables such as geographical features and economic structures may have an impact on the levels of these pollutants in the investigated PM_2.5_ samples.

**Table 1:** Descriptive Statistics for Detection Frequencies (DF, %) and Concentrations (pg/m^3^) of PPD Antioxidants and Their Quinone Derivatives (PPD-Qs) in PM_2.5_ from Chinese Cities. Site Roadside is Located at a Near-Street Point That is Also in City Guangzhou

	Guangzhou (*N* = 24)			roadside (*N* = 24)			Taiyuan (*N* = 24)		
compound	DF	median	range	DF	median	range	DF	median	range
*p*-Phenylenediamine Antioxidants (PPDs)									
IPPD	100	230	1.71–3690	100	661	2.11–2620	100	125	0.49–2830
CPPD	92	64.3	<MDL^a^-431	100	159	0.54–672	88	5.70	<MDL-27.5
6PPD	100	1820	22.2–6050	100	4040	2.23–9340	100	81.0	1.02–3190
7PPD	71	6.23	<MDL-18.7	88	7.66	MDL-12.3	46	NA^b^	<MDL-75.0
77PD	88	413	<MDL-2980	96	593	<MDL-1440	92	3.78	<MDL-4150
DPPD	100	553	55.0–2590	100	1250	0.77–2560	100	374	0.69–1940
DTPD	100	22.4	14.7–27.1	92	3.12	<MDL-3.49	83	3.23	<MDL-9.03
DNPD	50	16.3	<MDL-35.6	67	22.5	<MDL-61.3	58	5.22	<MDL-36.7
∑PPDs		3220	98.0–13,200		7990	10.9–14,200		1150	3.48–8630
*p*-Phenylenediamine Antioxidant Quinone Derivatives (PPD-Qs)									
IPPD-Q	75	65.5	<MDL-131	96	183	<MDL-3250	92	2220	<MDL-2940
CPPD-Q	79	12.4	<MDL-31.4	92	54.4	<MDL-1330	88	1280	<MDL-1380
6PPD-Q	100	1100	3.04–2350	100	2810	2.96–7250	100	744	2.44–1780
77PD-Q	100	527	0.57–2990	100	785	0.52–1050	92	11.3	<MDL-2870
DPPD-Q	92	41.5	<MDL-512	100	591	42.9–2100	88	552	<MDL-766
DTPD-Q	21	NA	<MDL-0.73	29	NA	<MDL-1.19	58	0.36	<MDL-3.23
∑PPD-Qs		1830	3.61–4490		6300	52.5–12,400		5040	5.59–8480

a MDL = method detection limit.

b NA = not available due to the low DF (<50%).

Since tire wear has been considered a contributor to the release of PPDs and 6PPD-Q in multiple environment matrices,^8,12,33,34^ we further investigate the occurrence of these novel PPD-Qs in the tire tread. As shown in Figure S4 and Table S6, a considerable level of PPD-Qs could be detected in the tire treads (0.12–78,800 ng/g), implying that tire rubber may be a potential source for these PPD-Qs in the PM_2.5_ samples. Based on these findings, we speculated that traffic intensity may also play a role in their concentrations in PM_2.5_ samples. Thus, we determined the levels of PPDs and PPD-Qs at a roadside site in Guangzhou (site roadside) and compared their abundance with a campus building site that was far away from heavy traffic jams (site Guangzhou). As indicated in Table 1 and Figure 2A, the ∑PPD-Qs at the roadside site (range of 52.5–12,400 pg/m^3^ and median of 6300 pg/m^3^) was found to be significantly higher (*p* < 0.001) than that at the campus building site (range of 3.61–4490 pg/m^3^ and median of 1830 pg/m^3^). Additionally, all the PPD-Qs at the roadside site exhibited higher median concentrations than their levels at the campus building site. Among them, the fold changes of three dominated PPD-Qs, including 6PPD-Q, 77PD-Q, and IPPD-Q, between the roadside site and campus building are 2.56, 1.49, and 2.79, respectively. Parallel evidence can be obtained by comparing the levels of their parent compounds between the two sampling sites, where the median of ∑PPDs at the roadside and campus sites was 7990 pg/m^3^ and 3220 pg/m^3^, respectively. It is reported that PPD antioxidants have been largely applied in commercial vehicle tire formulations (1–4% by mass).^11,35^ Fomba et al. found a larger contribution of tire wear to the atmosphere particulates at a traffic-dominated site (2.0–2.9%) compared to an urban background (1.7–2.1%).^36^ Similarly, Panko et al., have also observed a higher concentration of tire and road wear particles (TRWPs) in the air from the roadside (4 m from road, 16–32 ng/m^3^) compared to a relatively far site (10 m from road, 7–14 ng/m^3^).^37^ Our results are in line with these earlier studies and suggest that traffic intensity may be a possible factor affecting the emission of PPD-Qs to the environment.^7,13^

**Figure 3:**
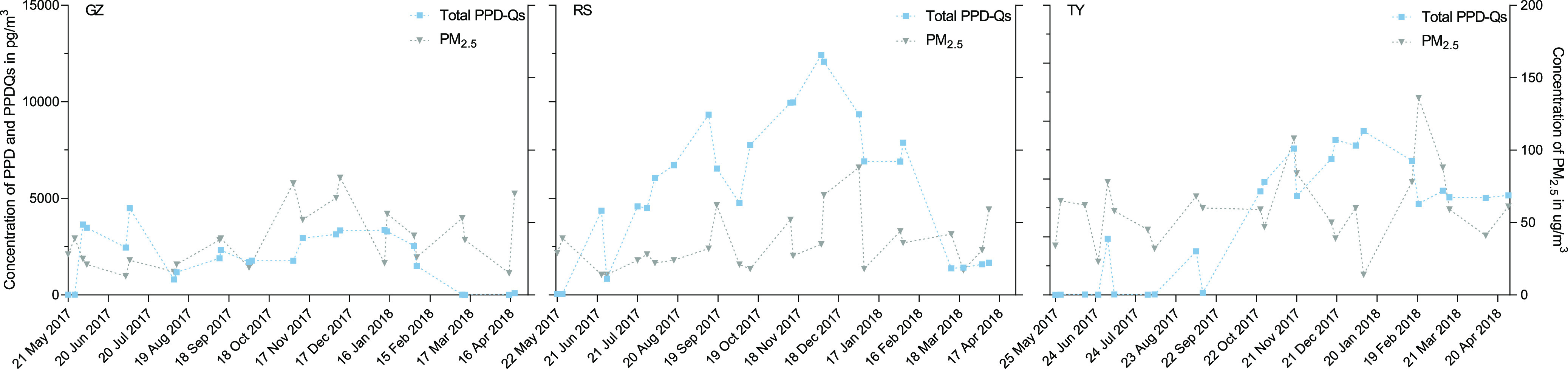
Temporal variation of PPD-Qs in PM_2.5_ at site Guangzhou (GZ), roadside of Guangzhou (RS), and Taiyuan (TY).

Apart from the geographical variability, the temporal and seasonal variations of PPD-Qs were also investigated. The time profiles of PPD-Qs in PM_2.5_ samples between the two megacities from May 2017 to April 2018 are displayed in Figure 3. It was intriguing to note that the highest values of ∑PPD-Qs detected in Guangzhou and Taiyuan were in July (4490 pg/m^3^) and January (8480 pg/m^3^), respectively. A temporary augment of ∑PPD-Qs (2900 pg/m^3^) was also observed in Taiyuan in July, 2017. As for the roadside site, we noticed a consistent increase in the ∑PPD-Qs from October to December 2017. As an on-site sampling point, atmospheric particles collected from the roadside site may be more susceptible to the emission of tire wear particles and directly reflect the release of PPD-Qs and their parent compounds. By observing their seasonal variations (Figure S6), it is clear that both Guangzhou and Taiyuan showed distinct variation patterns of PPD-Qs. Taiyuan demonstrated a decrease–increase trend with clear peak and bottom values observed in the winter and summer, respectively, while Guangzhou illustrated a fluctuant trend with slightly higher levels of PPD-Qs in both summer and winter. On the other hand, the levels of PPD-Qs at the roadside site were shown to be generally high from summer to winter.

### Correlations Analysis between PPD-Qs and PPDs

**Figure 4:**
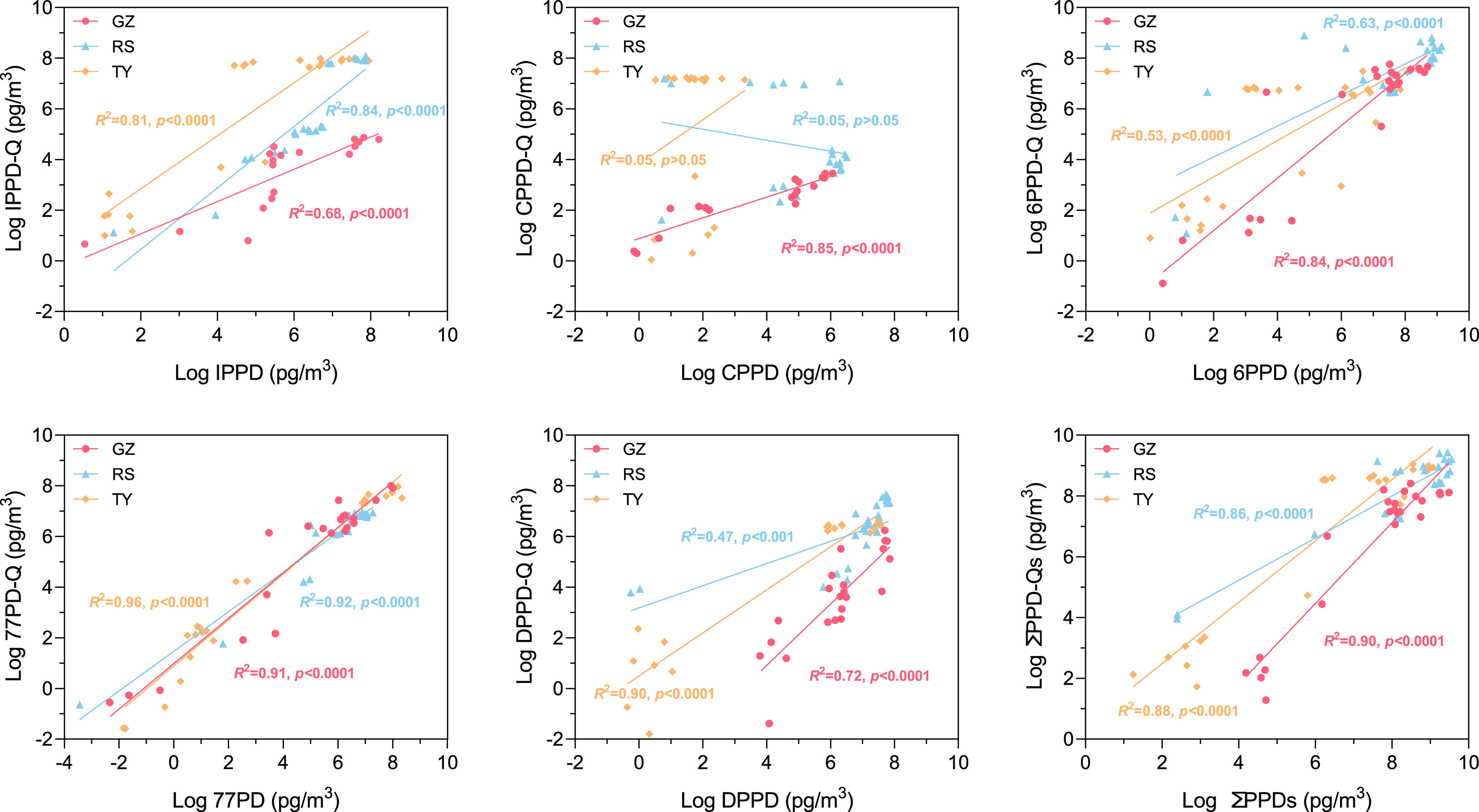
Linear regressions between each PPD-Qs and their parent PPDs and correlation between their total concentrations at different sites of China.GZ: site Guangzhou, RS: site Roadside in Guangzhou, and TY: site Taiyuan. Logarithm of data to base 10 (log_10_).

To further elucidate the potential commonalities in the source of these PPD-Qs in the environment, their Spearman correlation analysis with their parent PPDs in PM_2.5_ from different sites was characterized (Figure S7). Significant positive correlations among these PPD-Qs and PPDs in Guangzhou (71 pairs, *r* = 0.41–0.95 and *p* < 0.05), roadside (62 pairs, *r* = 0.41–0.98 and *p* < 0.05), and Taiyuan (72 pairs, *r* = 0.41–0.97 and *p* < 0.05) were observed, which implied some common emission sources and/or similar environmental fates of PPDs and PPD-Qs. Similar to 6PPD-Q, 77PD-Q also illustrated strong correlations with other PPDs, especially for CPPD (*r* = 0.52–0.90 and *p* < 0.01), 6PPD (*r* = 0.55–0.94 and *p* < 0.01), and 7PPD (*r* = 0.89–0.94 and *p* < 0.01). To delve into the relationship between individual PPD-Qs and PPDs, their linear regressions were performed as shown in Figures 4 and S8. Most of the PPD-Qs were shown to have a high degree of convergence with their parent PPDs among different sites, especially 6PPD-Q/6PPD (*R*^2^ = 0.53–0.84 and *p* < 0.0001), 77PD-Q/77PD (*R*^2^ = 0.91–0.96 and *p* < 0.0001), IPPD-Q/IPPD (*R*^2^ = 0.68–0.84 and *p* < 0.0001), and the total PPD-Qs to the total PPDs (*R*^2^ = 0.86–0.90 and *p* < 0.0001). These results may imply that most of these quinones were transformed from their parent compounds like 6PPD/6PPD-Q.^13^ Additionally, we found that PPDs and PPD-Qs showed unified higher regression coefficients (*R*^2^ = 0.68–0.91) in Guangzhou compared to the Taiyuan city, especially for CPPD-Q/CPPD (*R*^2^ = 0.85 and *p* < 0.0001), 6PPD-Q/6PPD (*R*^2^ = 0.84 and *p* < 0.0001), and ΣPPD-Qs/ΣPPDs (*R*^2^ = 0.90 and *p* < 0.05). Comparatively, dis-convergence was found in Taiyuan like CPPD-Q/CPPD (*R*^2^ = 0.05 and *p* > 0.05) and DTPD-Q/DTPD (*R*^2^ = 0.01 and *p* > 0.05). Many factors including geographical and economic character, the source and physicochemical properties of atmospheric particulates, and the preference for the use of rubber products in the two megacities could confound these observations.^17^ More research based on specific measurements is still needed to confirm their origination due to the deficiency in the formation mechanisms of these PPD-Qs.

### Toxicological Implications

Given the prevalence of these novel PPD-Qs and their parent compounds in respirable fine particulate matter, assessing their exposure to humans is essential. Here, the estimated daily intakes (EDIs) of these contaminants for different subpopulation groups including children, resident adults, and occupational workers under median (calculated with geometric mean) and high (calculated with 95th percentile) exposure scenarios were assessed (Table S7). For different subpopulation groups under different exposure scenarios, the EDIs of these PPD-Qs ranged from 0.16 to 1.25 ng kg_bw_^–1^ day^–1^, while their parent PPDs were in the range of 0.19–1.41 ng kg_bw_^–1^ day^–1^. These comparable exposure levels suggest an important but overlooked exposure caused by these PPD-derived quinones. In the median scenario, the total EDI of PPD-Qs and PPDs for children, resident adults, and workers are 0.36, 0.44, and 0.75 ng kg_bw_^–1^ day^–1^, respectively, while these values in the high scenario are increased to 1.27, 1.56, and 2.66 ng kg_bw_^–1^ day^–1^, respectively. These levels were comparable to those of the recently reported plastic additives like tris(2-chloroisopropyl) phosphate (0.14–5.7 ng kg_bw_^–1^ day^–1^), diethyl phthalate (0.28–3.7 ng kg_bw_^–1^ day^–1^), and bis(2-ethylhexyl) adipate (0.17–1.9 ng kg_bw_^–1^ day^–1^) in the PM_2.5_ of the Pearl River Delta in South China.^38^ Compared to the EDI through inhalation of other PM_2.5_-bonded rubber additives like BTHs assessed in Guangzhou (35–254 10^–3^ ng kg_bw_^–1^ day^–1^), Shanghai (26.7–226 10^–3^ ng kg_bw_^–1^ day^–1^), Taiyuan (21.1–228 10^–3^ ng kg_bw_^–1^ day^–1^), and Tianjin (2.76–4.89 10^–3^ ng kg_bw_^–1^ day^–1^), the exposure of these PPDs and PPD-Qs were 1–2 magnitudes higher due to their relatively higher concentrations.^32,39^

For different population groups, it is clear that the occupational workers under the high scenario showed the highest exposure amount than the other groups, with their total EDI (2.66 ng kg_bw_^–1^ day^–1^) being almost six times greater than that of the ordinary resident adults under the median scenario (0.44 ng kg_bw_^–1^ day^–1^). These results indicate that the laborers who have high-frequency contact with the roadside ambient air like street cleaner or traffic police may pay more attention to the potential adverse effects caused by these novel contaminants. Although the inhalation rate and exposure frequency of children were lower than those of the adults, their total EDI was shown comparable in both median (0.36/0.44 ng kg_bw_^–1^ day^–1^) and high scenarios (1.27/1.56 ng kg_bw_^–1^ day^–1^) due to their relatively lower body weight. These EDI results of PPDs and their derivatives through ambient air inhalation are comparable to the evaluated doses of other widely used synthetic additives and their transformation products like amino antioxidants (0.02–0.48 ng kg_bw_^–1^ day^–1^) and phenolic antioxidants (0.2–124 ng kg_bw_^–1^ day^–1^) through dust ingestion.^6,40^ This implies that both ambient air inhalation and dust ingestion are important exposure pathways for these antioxidants as well as their derivatives.

The identification and determination of a series of emerging PPD antioxidant-derived quinones in PM_2.5_ implies that our current understanding of airborne contaminants is still limited. Besides the newly identified 77PD-Q and other PPDs/PPD-Qs, numerous anthropogenic products and their transformation products may also occur in the PM_2.5_, emphasizing the significance of novel contaminant screening. Although these samples were well-stored, transformation and degradation of the target compounds could have also happened, which may have affected their concentrations. Our findings reveal the overlooked exposure caused by the coexistence of various transformation products along with their parent chemicals. Different environmental scenarios shall complicate both the levels and composition patterns of the contaminants, which need detailed investigations. This will further improve our understanding of the sources and toxicity of PM_2.5_.

An increasing number of studies have focused on the toxicity of these PPD antioxidants.^8−10,15,33^ According to the European Chemical Agency, several of them were labeled as very toxic to aquatic life with long-lasting effects and harmful if swallowed and may cause an allergic skin reaction.^33^ Considering their use for protecting rubber products from being oxidized, their ozonized PPD-Qs are likely to be more stable in the environment. Although the adverse effects of such PPD-Qs were not clearly unveiled and comprehensively assessed, there is some evidence of the toxicity of 6PPD-Q to aquatic organisms.^15^ Besides coho salmon, 6PPD-Q was reported to have acute toxicity to brook trout (24 h) and rainbow trout (72 h) with LC_50_s of 0.59 and 1.00 μg/L, respectively.^41^ Similarly, other antioxidant-derived quinone transformation products like 2,6-di-*tert*-butyl-1,4-benzoquinone (BHT-Q) were also proved to cause DNA damage and apoptosis at a concentration as low as 1 μM.^42,43^ In analogy to other types of PM_2.5_-bounded quinones that have been found to cause oxidative stress and DNA toxicity,^44^ these PPD-Qs could also lead to similar outcomes. Given the prolonged exposure of these antioxidants and their quinone derivatives, especially to traffic-related occupations, further studies are expected to assess the toxicity and mine the noxious mechanism of these derivatives.

## Supporting Information Available

Synthesis and characterization of 77PD-Q; exposure assessment of PPDs and PPD-Qs; measurement of PPD-Qs; instrument parameters; optimized MRM parameters, recoveries, matrix effect, MQLs, and MDLs of the analytes; estimated daily intake; structures and abbreviations for the PPDs and PPD-Qs; ^1^H NMR (upper) and ^13^C NMR spectra; specific geographical distribution of sampling site; MRM chromatograms of the six analyzed PPD-Qs; specific fragmentation pathways; seasonal concentration variation; Spearman correlation coefficients; and linear regression (PDF)
