# Supplementary material for: Beyond Substituted p-Phenylenediamine Antioxidants: Prevalence of Their Quinone Derivatives in PM2.5
Source: Environ Sci Technol. 2022 Jul 14;56(15):10629–37. doi: 10.1021/acs.est.2c02463 (PMC9393868; doi:10.1021/acs.est.2c02463)
Supplement: Supplementary file 1 — es2c02463_si_001.pdf [file es2c02463_si_001.pdf]

Supporting Information

Beyond substituted *p*-phenylenediamine antioxidants: Prevalence of their quinone derivatives in $PM_{2.5}$

Wei Wang[a,1], Guodong Cao[a,1], Jing Zhang[a], Pengfei Wu[a], Yanyan Chen[a], Zhifeng Chen[b], Zenghua Qi[b], Ruijin Li[c], Chuan Dong[c], and Zongwei Cai[a,*]

[a] *State Key Laboratory of Environmental and Biological Analysis, Department of Chemistry, Hong Kong Baptist University, Hong Kong SAR, China*

[b] *School of Environmental Science and Engineering, Guangdong University of Technology, Guangzhou 510006, China*

[c] *Institute of Environmental Science, Shanxi University, Taiyuan 030006, China*

Number of pages: 23

Number of figures: 8

Number of tables: 7

[*] Corresponding author: Zongwei Cai, email: zwcai@hkbu.edu.hk

[1] These authors contributed equally to this work.

Contents

Text S1. Synthesis and characterization of 77PD-quinone (77PD-Q) S3

Text S2. Exposure assessment of PPDs and PPD-Qs S4

Text S3. Measurement of PPD-Qs in the tire treads S5

Table S1. Information on analyte name, abbreviations, CAS No and SMILES S6

Table S2. Sampling, atmosphere quality information and meteorological condition. S7

Table S3. Detailed instrument parameters for identification and quantification............................. S9

Table S4. Optimized MRM parameters, recoveries, MDLs/MQLs of the analytes S10

Table S5. Parameters used to estimate human exposure to PPDs and PPD-Qs S11

Table S6. Measured concentration of PPD-Qs in tire treads.. S12

Table S7. Estimated daily intake rates of PPDs, PPD-Qs S13

Figure S1. Structures and abbreviations for the PPD and PPD-Qs S14

Figure S2. ^{1}H NMR and ^{13}C NMR spectra of 77PD-Q S15

Figure S3. Specific geographical distribution of each sampling site S16

Figure S4. MRM chromatograms of PPD-Qs in standards, $PM_{2.5}$ samples and tire tread. S17

Figure S5. Specific fragmentation pathways for each PPD-Qs.. S19

Figure S6. Seasonal variation of PPDs and PPD-Qs each site S20

Figure S7. Spearman correlation coefficients among individual PPD and PPD-Qs. S21

Figure S8. Linear regression between the concentration of DTPD-Q with DTPD S22

References... S23

Text S1. Synthesis and characterization of 77PD-quinone (77PD-Q)

1,4-benzoquinone (300 mg, 2.8 mmol) was dissolved in methanol (10 mL) in a round-bottomed flask (25 mL). A mixture of 2-amino-5-methylhexane (280 µL, 1.85 mmol) in acetic acid (72 µL, 1.3 mmol) and methanol (5 mL) was added drop by drop. The mixture was stirred for 3 h at room temperature. After completion of the reaction, the crude product was filtered, dried under reduced pressure, and purified by chromatography (*n*-hexane/ dichloromethane = 2:1 to 1:1) with silica gel (200-300 mesh, Qingdao Marine Chemical Factory, China) to afford 77PD-quinone (103 mg, 0.31 mmol). The compound was characterized as: ^{1}H NMR (400 MHz, $CDCl_3$): δ 6.53 (s, broad, 2H), 5.29 (s, 2H), 3.40 (m, 2H), 1.54 (m, 6H), 1.20 (d, J = 6.4 Hz, 6H), 1.18 (m, 4H), 0.86 (d, J = 6.8 Hz, 12H). ^{13}C NMR (400 MHz, $CDCl_3$): δ 178.1, 150.1, 92.7, 48.9, 35.1, 34.2, 28.1, 22.6, 22.5, 19.9 (Figure S2). IR (neat): 3218, 2956, 2928, 2872, 1636, 1560, 1488, 1451, 1358, 1293, 1235, 1149, 1122, 958, 816, 729 cm^{-1}; HR-ESI-MS: *m/z* = 335.2683 $[C_{20}H_{35}N_2O_2]^+$ (calcd *m/z* = 335.2693). HR-ESI-MS/MS (*m/z*) 237.1592 $[M - C_7H_{14}]^+$ (calcd *m/z* = 237.1598), 167.0813 $[M - C_{12}H_{24}]^+$ (calcd *m/z* = 167.0815), 139.0499 $[M - C_{14}H_{28}]^+$ (calcd *m/z* = 139.0502), 97.1014 $[M - C_{13}H_{22}N_2O_2]^+$ (calcd *m/z* = 97.1012) (Figure S4).

Text S2. Exposure assessment of PPDs and PPD-Qs.

The estimated daily intake (EDI, in ng kg_{bw}^{-1} day^{-1}) of humans to PPDs and PPD-Qs via ambient inhalation was calculated with the following equation:

$$EDI = \frac{C_{AP} \times IR \times ET \times EF \times ED}{BW \times AT} \quad (1)$$

where EDI is the average daily dose for each compound via ambient inhalation in ng kg_{bw}^{-1} day^{-1}, C_{AP} is the total concentration of the determined contaminants in ng m^{-3}, IR and ET are the inhalation rate (m^3 day^{-1}) of air particles and exposure time (h day^{-1}). EF is the exposure frequency (days $years^{-1}$) while ED is the exposure duration (years). BW and AT are the body weight (kg) and average time during exposure (days), respectively. The parameters used to evaluate the EDI for children and adults were according to the risk assessment guidance[1-3] and relative studies[4-6] as shown in Table S5. Human exposure to each kind of contaminant was estimated using the sum of concentrations for each group in each microenvironment. Different subpopulation groups, classified according to age (children and adults) and identity (residents and workers) were taken into account. Moreover, considering the obtained concentrations, two exposure scenarios including median (based on geometric mean) and high (based on 95th percentile) were simulated.

Text S3. Measurement of PPD-Qs in the tire treads.

Six tire tread samples were collected from an auto repair shop, with the tires showing a varying oxidation level that has been used for 0.6-4 years. The pretreatment of these tire treads was following our early work.[6] A varying mass of tire treads (65.7-106.5 mg) was extracted with dichloromethane (1.5 mL twice) and acetonitrile (2 mL once) for 30 minutes each. The extracts were nitrogen purged to near dryness and redissolved in acetonitrile for 1 mL with 20 ng internal standard was spiked. The analysis approach is consistent with the determination of $PM_{2.5}$ samples. Triplet analysis of each sample was performed and the results were shown as mean ± standard deviation (S.D.) in Table S5.

Table S1. Information on analyte name, abbreviations, CAS No and SMILES.

Compound name	Abbreviation	CAS No.	SMILES
N-isopropyl-m'-phenyl-p-phenylenediamine	IPPD	101-72-4	N(c(ccc(Nc(cccc1)c1)c2)c2)C(C)C
N-phenyl-N'-cyclohexyl-p-phenylenediamine	CPPD	101-87-1	HC1C(H)C(H)C(H)C(H)C1Nc1cc(Nc2ccccc2)ccc1
N-1, 3-dimethylbutyl-n'-phenyl-p-phenylenediamine	6PPD	793-24-8	HC(H)C(C(H)H)C(H)C(C(H)H)Nc1cc(Nc2ccccc2)ccc1
(1,4-dimethylpentyl)-N'-phenylbenzene-1,4-diamine	7PPD	3081-01-4	CC(C)CCC(C)Nc1ccc(cc1)Nc2ccccc2
N,N'-Bis(1,4-dimethylpentyl)-P-phenylenediamine	77PD	3081-14-9	CC(C)CCC(C)Nc1ccc(cc1)NC(C)CCC(C)C
N,N'-diphenyl-p-phenylenediamine	DPPD	74-31-7	c1(Nc2ccccc2)ccc(Nc2ccccc2)cc1
N,N''-Bis(methylphenyl)-1,4-benzenediamine	DTPD	15017-02-4	HC(H)c1c(Nc2ccc(Nc3c(C(H)H)cccc3)cc2)cccc1
N,N'-Di-b-naphthyl-p-phenylenediamine	DNPD	93-46-9	c1ccc2cc(ccc2c1)Nc3ccc(cc3)Nc4cc5ccccc5cc4
2-(isopropylamino)-5-(phenylamino)cyclohexa-2,5-diene-1,4-dione	IPPD-Q	NA	HC(H)C(C(H)H)NC1C(=O)C=C(Nc2ccccc2)C(=O)C=1
2-(cyclohexylamino)-5-(phenylamino)cyclohexa-2,5-diene-1,4-dione	CPPD-Q	NA	HC1C(H)C(H)C(H)C(H)C1NC1C(=O)C=C(Nc2ccccc2)C(=O)C=1
2-anilino5-[(4-methylpentan-2-yl)amino]cyclohexa-2,5diene-1,4-dione	6PPD-Q	NA	HC(H)C(C(H)H)C(H)C(C(H)H)NC1C(=O)C=C(Nc2ccccc2)C(=O)C=1
2,5-bis((5-methylhexan-2-yl)amino)cyclohexa-2,5-diene-1,4-dione	77PD-Q	NA	C1(NC(C)CCC(C)C)C(=O)C=C(NC(C)CCC(C)C)C(=O)C=1
2,5-bis(phenylamino)cyclohexa-2,5-diene-1,4-dione	DPPD-Q	NA	C1(Nc3ccccc3)C(=O)C=C(Nc2ccccc2)C(=O)C=1
2,5-bis(o-tolylamino)cyclohexa-2,5-diene-1,4-dione	DTPD-Q	NA	HC(H)c1c(NC2C(=O)C=C(Nc3c(C(H)H)cccc3)C(=O)C=2)cccc1

Table S2. Date of sampling, atmosphere quality parameters ($PM_{2.5}$ and ozone) and meteorological condition (temperature and weather) during the sampling period. The atmosphere quality parameters and meteorological condition data were based on the local monitoring database from China national urban air quality real-time publishing platform and National Meteorological Information Center[7, 8]. GZ: Site Guangzhou; RS: Site roadside; TY: Site Taiyuan.

Site No.	Date	$PM_{2.5}$ (μg m^{-3})	O_3 (μg m^{-3})	Temperature (°C)	Humidity (%)	Wind Speed (m/h)	Weather
GZ-1	2017/5/21	28	116	25.2	87.2	8.3	Rainy
GZ-2	2017/5/26	39	107	25.4	63.6	10.1	Cloudy
GZ-3	2017/6/1	25	106	31.0	68.9	15.0	Rainy
GZ-4	2017/6/4	21	99	29.2	85.8	9.2	Rainy
GZ-5	2017/7/3	13	19	26.1	90.3	9.2	Rainy
GZ-6	2017/7/6	24	61	28.2	81.2	11.0	Rainy
GZ-7	2017/8/8	16	111	32.0	66.3	11.8	Rainy
GZ-8	2017/8/10	21	78	31.1	72.7	12.3	Rainy
GZ-9	2017/9/11	38	171	29.9	73.1	5.5	Rainy
GZ-10	2017/9/12	39	129	31.1	66.9	11.7	Cloudy
GZ-11	2017/10/3	19	110	31.4	66.7	8.3	Rainy
GZ-12	2017/10/5	22	103	28.5	76.1	7.3	Rainy
GZ-13	2017/11/5	77	162	22.2	46.3	17.4	Cloudy
GZ-14	2017/11/12	52	90	25.0	71.0	7.7	Cloudy
GZ-15	2017/12/7	67	28	17.9	64.5	7.9	Cloudy
GZ-16	2017/12/10	81	49	16.3	47.2	8.9	Cloudy
GZ-17	2018/1/12	22	79	12.1	29.6	16.9	Sunny
GZ-18	2018/1/14	56	94	14.3	54.9	6.1	Cloudy
GZ-19	2018/2/3	41	101	10.3	35.6	20.4	Cloudy
GZ-20	2018/2/5	26	79	9.2	25.0	21.0	Sunny
GZ-21	2018/3/11	53	172	17.9	57.3	4.6	Cloudy
GZ-22	2018/3/13	38	118	21.9	64.1	6.9	Rainy
GZ-23	2018/4/15	15	44	17.7	76.4	17.6	Rainy
GZ-24	2018/4/19	70	164	23.0	77.9	3.8	Rainy
RS-1	2017/5/22	29	65	25.1	87.8	6.2	Rainy
RS-2	2017/5/26	39	107	25.4	63.6	10.1	Cloudy
RS-3	2017/6/24	14	89	30.0	75.9	8.8	Rainy
RS-4	2017/6/28	14	104	30.1	69.7	9.8	Rainy
RS-5	2017/7/21	24	79	29.3	76.6	8.3	Cloudy
RS-6	2017/7/28	28	180	32.3	63.3	6.0	Cloudy
RS-7	2017/8/3	22	50	27.5	87.5	7.4	Rainy
RS-8	2017/8/17	24	148	31.0	66.0	6.0	Sunny
RS-9	2017/9/12	32	129	31.1	66.9	11.7	Cloudy

RS-10	2017/9/18	62	261	30.2	58.7	8.5	Sunny
RS-11	2017/10/5	21	103	28.5	76.1	7.3	Rainy
RS-12	2017/10/13	18	112	24.8	59.7	24.3	Sunny
RS-13	2017/11/12	52	90	25.0	71.0	7.7	Cloudy
RS-14	2017/11/14	27	22	22.6	90.6	4.9	Rainy
RS-15	2017/12/5	35	57	17.1	61.4	15.6	Cloudy
RS-16	2017/12/7	69	28	17.9	64.5	7.9	Cloudy
RS-17	2018/1/2	88	64	19.1	65.5	6.3	Cloudy
RS-18	2018/1/6	18	5	12.3	95.3	6.9	Rainy
RS-19	2018/2/2	44	61	9.0	55.8	17.9	Sunny
RS-20	2018/2/4	36	81	8.5	30.3	21.5	Cloudy
RS-21	2018/3/12	42	121	19.8	59.2	5.1	Cloudy
RS-22	2018/3/21	17	100	16.6	52.0	18.4	Sunny
RS-23	2018/4/4	31	134	25.5	63.3	7.7	Cloudy
RS-24	2018/4/9	59	154	21.0	60.3	7.7	Sunny
TY-1	2017/5/25	34	140	22.6	33.4	8.1	Sunny
TY-2	2017/5/29	65	252	25.5	44.7	18.3	Sunny
TY-3	2017/6/16	62	253	25.1	40.2	5.9	Cloudy
TY-4	2017/6/26	23	181	22.9	56.8	6.0	Sunny
TY-5	2017/7/3	78	254	28.0	51.9	7.8	Rainy
TY-6	2017/7/8	58	259	28.6	48.3	9.2	Sunny
TY-7	2017/8/2	45	136	25.4	67.7	8.5	Rainy
TY-8	2017/8/7	32	94	24.2	60.8	5.9	Sunny
TY-9	2017/9/7	68	155	21.3	62.2	6.5	Sunny
TY-10	2017/9/12	60	149	21.0	57.1	5.6	Cloudy
TY-11	2017/10/25	59	29	10.6	86.3	4.3	Rainy
TY-12	2017/10/28	47	42	8.1	68.6	10.8	Sunny
TY-13	2017/11/19	108	39	-1.3	47.8	4.7	Sunny
TY-14	2017/11/21	84	73	1.6	45.7	12.9	Sunny
TY-15	2017/12/17	50	56	-5.4	48.1	8.3	Sunny
TY-16	2017/12/20	39	64	-0.3	38.8	9.2	Cloudy
TY-17	2018/1/4	60	28	-4.2	75.0	10.5	Snowy
TY-18	2018/1/10	14	79	-3.8	38.6	18.1	Sunny
TY-19	2018/2/15	78	72	-1.3	33.0	5.8	Sunny
TY-20	2018/2/20	136	94	0.7	50.8	13.8	Cloudy
TY-21	2018/3/10	88	106	8.6	39.9	13.2	Sunny
TY-22	2018/3/15	59	77	9.45	42.5	21.7	Sunny
TY-23	2018/4/11	41	99	15.6	24.5	8.1	Cloudy
TY-24	2018/4/28	61	179	21.2	42.2	7.3	Cloudy

Table S3 Instrument parameters used in the identification and quantification of the analytes.

Instrument	Parameter	Value
UPLC	Flow rate	300 μL/min
	Injection volume	2 uL
	Column	Waters Acquity HSS T3 column (1.8 μm, 2.1×100 mm)
	Column temperature	35 °C
	Mobile phase	(A) deionized water with 0.1% formic acid in (B) acetonitrile with 0.1% formic acid
	Solvent gradient	2% B for 1 min, increased linearly to 100% B in 19 min and hold for 3 min, decreased to 2% B in 0.1 min and hold for 4.9 min.
ESI-HR MS	Ionization mode	Positive ESI
	Spray voltage	3500 V
	Capillary voltage	320 °C
	Scan mode	dd-MS2
	Collision energy	10, 20, 40 ev
	Scan range	200-400 m/z
ESI-TQ MS	Ionization mode	Positive ESI
	Vaporizer temperature	350 °C
	Collision energy	Specified for each analyte (Table S4)

Table S4 Optimized MRM parameters, recoveries (mean ± S.D.）, matrix effect, MQLs and MDLs of the analytes.

Compound	Precursor ion	Quantifier product ion (m/z)	Collision energy (V)	Qualifier product ion (m/z)	Blank recovery (%)	Matrix recovery (%)	Matrix effect[a] (%)	MQLs[b] (pg/m^3)	MDLs[b] (pg/m^3)
IPPD	227.2	184.1	26	212.1/168.1	96±5	89±6	97±5	0.05	0.01
CPPD	267.2	185.1	22	223.1/130.1	91±1	79±2	82±2	0.15	0.05
6PPD	269.2	93.1	32	184.1/212.1	80±1	77±3	81±3	0.25	0.07
7PPD	283.2	184.1	20	93.1/212.1	72±4	70±2	82±2	0.09	0.03
77PD	305.3	206.2	16	233.2/135.1	74±2	77±3	114±6	0.05	0.02
DPPD	261.1	184.1	26	169.1/107.1	77±1	74±3	80±1	0.34	0.10
DTPD	289.2	198.1	23	183.1/106.1	78±3	71±2	87±2	1.66	0.50
DNPD	361.2	234.1	26	219.1/142.1	93±1	97±4	101±5	0.19	0.06
IPPD-Q	257.1	187.1	24	215.1/172.1	90±2	95±2	103±4	0.21	0.06
CPPD-Q	297.2	187.1	28	215.1/98.1	79±2	87±3	75±3	0.20	0.06
6PPD-Q	299.2	241.1	26	215.1/187.1	80±2	84±2	82±4	0.08	0.02
77PD-Q	335.2	237.1	18	139.0/97.1	115±1	93±5	78±1	0.13	0.04
DPPD-Q	291.1	263.1	20	235.1/144.1	81±1	89±4	111±6	0.03	0.01
DTPD-Q	319.1	184.1	27	212.1/301.1	90±3	84±2	89±2	0.12	0.04

[a] Matrix effects are evaluated as the ratios between the signals of target chemicals analyzed in the spiked matrix and the chemical signals measured for the same concentrations in ACN.

[b] MQL/MDL calculated for a sampling volume of 126 m^3 and taking into account the method recoveries.

Table S5. Parameters used to estimate human exposure to PPDs and PPD-Qs.[1-6]

Parameters	Children	Adults	
		Residents	Occupational workers
Inhalation rate (IR_{inh}, m^3/day)	7.60	16.7	16.7
Body weight (BW, kg)	16.6	63.0	63.0
Exposure frequency (EF, days/year)	365	365	365
Exposure duration (ED, years)	6	24	24
Average time during exposure (AT, day)	365×ED	365×ED	365×ED
Exposure time (ET, h/day)	2.2	4.7	8

Table S6. Measured concentration (ng/g, mean ± S.D.) of PPD-Qs in tire treads.

No.	IPPD-Q	CPPD-Q	6PPD-Q	77PD-Q	DPPD-Q	DTPD-Q
TT-1	6.56±0.71	131±2.86	8790±272	0.45±0.13	95.4±3.21	2.28±1.32
TT-2	70.8±4.25	268±85.1	77600±1270	5.12±0.70	199±2.98	52.7±4.35
TT-3	6.91±0.41	153±12.9	9270±502	0.12±0.01	95.6±5.84	1.20±0.16
TT-4	63.9±2.12	2370±63.8	78800±31.7	1.02±0.13	241±1.59	66.7±8.60
TT-5	101±5.89	2690±62.4	76200±260	1.66±0.03	275±12.2	75.0±3.04
TT-6	66.9±1.87	2040±38.5	58900±777	0.90±0.01	244±8.87	62.9±2.89

Note: Semi-quantitative results without evaluation of matrix effects and recoveries.

Table S7. Estimated daily intake of PPDs, PPD-Qs, and their total amount via ambient inhalation for children, resident adults, and occupational workers in different exposure scenarios.

Compounds	Median scenario			High scenario		
	Children	Adults		Children	Adults	
		Residents	Workers		Residents	Workers
IPPD	3.10E-02	3.83E-02	6.53E-02	1.08E-01	1.34E-01	2.28E-01
CPPD	5.30E-03	6.55E-03	1.12E-02	2.32E-02	2.86E-02	4.87E-02
6PPD	9.16E-02	1.13E-01	1.93E-01	3.13E-01	3.87E-01	6.58E-01
7PPD	3.51E-04	4.34E-04	7.39E-04	2.01E-03	2.48E-03	4.22E-03
77PD	2.57E-02	3.17E-02	5.40E-02	1.23E-01	1.52E-01	2.59E-01
DPPD	3.94E-02	4.87E-02	8.29E-02	1.00E-01	1.24E-01	2.11E-01
DTPD	3.90E-04	4.81E-04	8.19E-04	1.05E-03	1.29E-03	2.20E-03
DNPD	4.13E-04	5.11E-04	8.69E-04	1.68E-03	2.07E-03	3.52E-03
ΣPPDs	1.94E-01	2.40E-01	4.08E-01	6.73E-01	8.31E-01	1.41E+00
IPPD-Q	3.57E-02	4.42E-02	7.52E-02	1.24E-01	1.54E-01	2.62E-01
CPPD-Q	1.59E-02	1.97E-02	3.35E-02	5.74E-02	7.09E-02	1.21E-01
6PPD-Q	6.41E-02	7.92E-02	1.35E-01	2.36E-01	2.92E-01	4.97E-01
77PD-Q	2.79E-02	3.44E-02	5.86E-02	1.02E-01	1.26E-01	2.15E-01
DPPD-Q	1.72E-02	2.13E-02	3.62E-02	7.37E-02	9.10E-02	1.55E-01
DTPD-Q	1.19E-05	1.47E-05	2.50E-05	6.19E-05	7.65E-05	1.30E-04
ΣPPD-Qs	1.61E-01	1.99E-01	3.38E-01	5.94E-01	7.34E-01	1.25E+00
Total	3.55E-01	4.39E-01	7.47E-01	1.27E+00	1.56E+00	2.66E+00

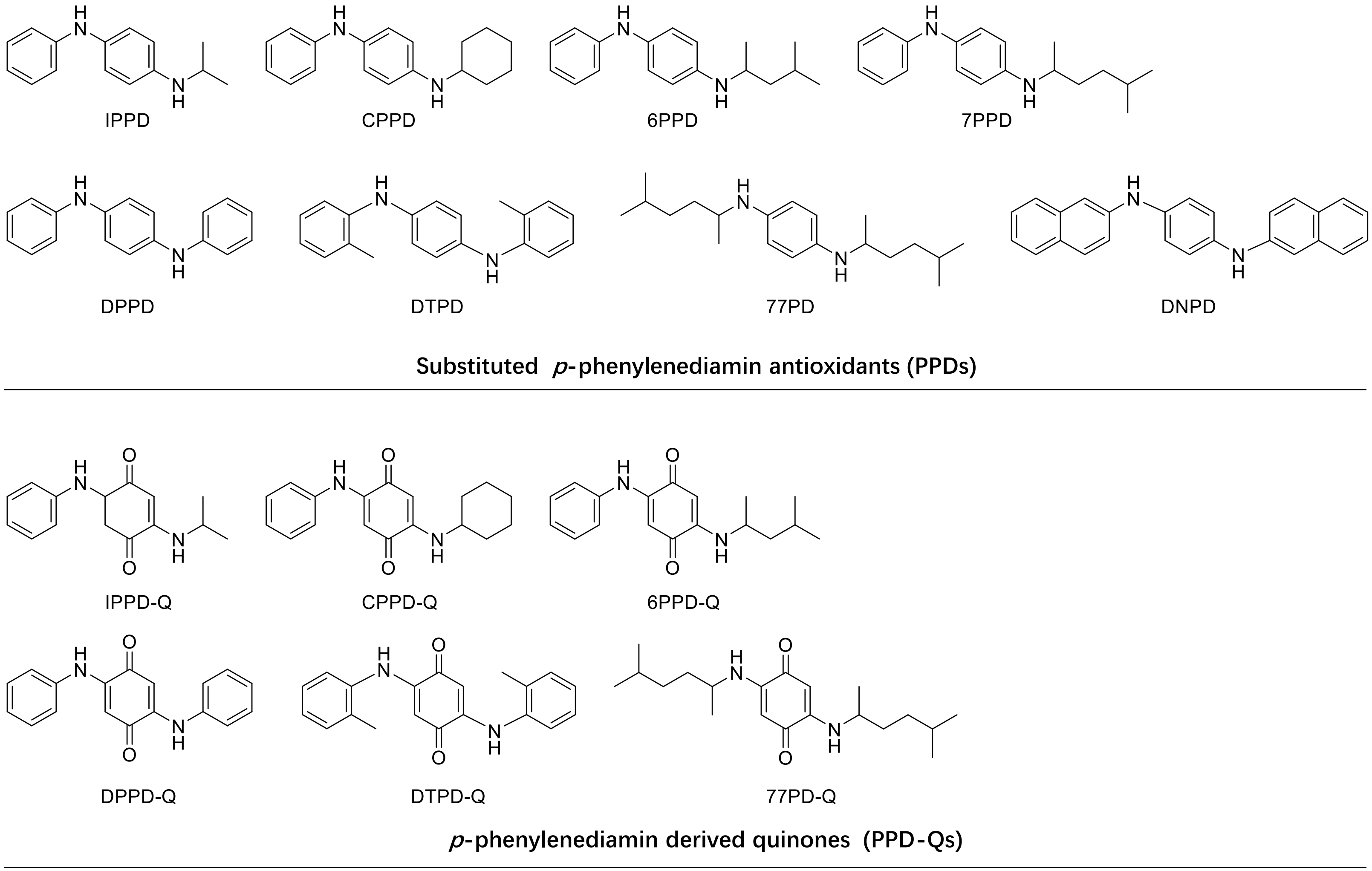

Figure S1. Structures and abbreviations for the PPDs and PPD-Qs discussed in this study.

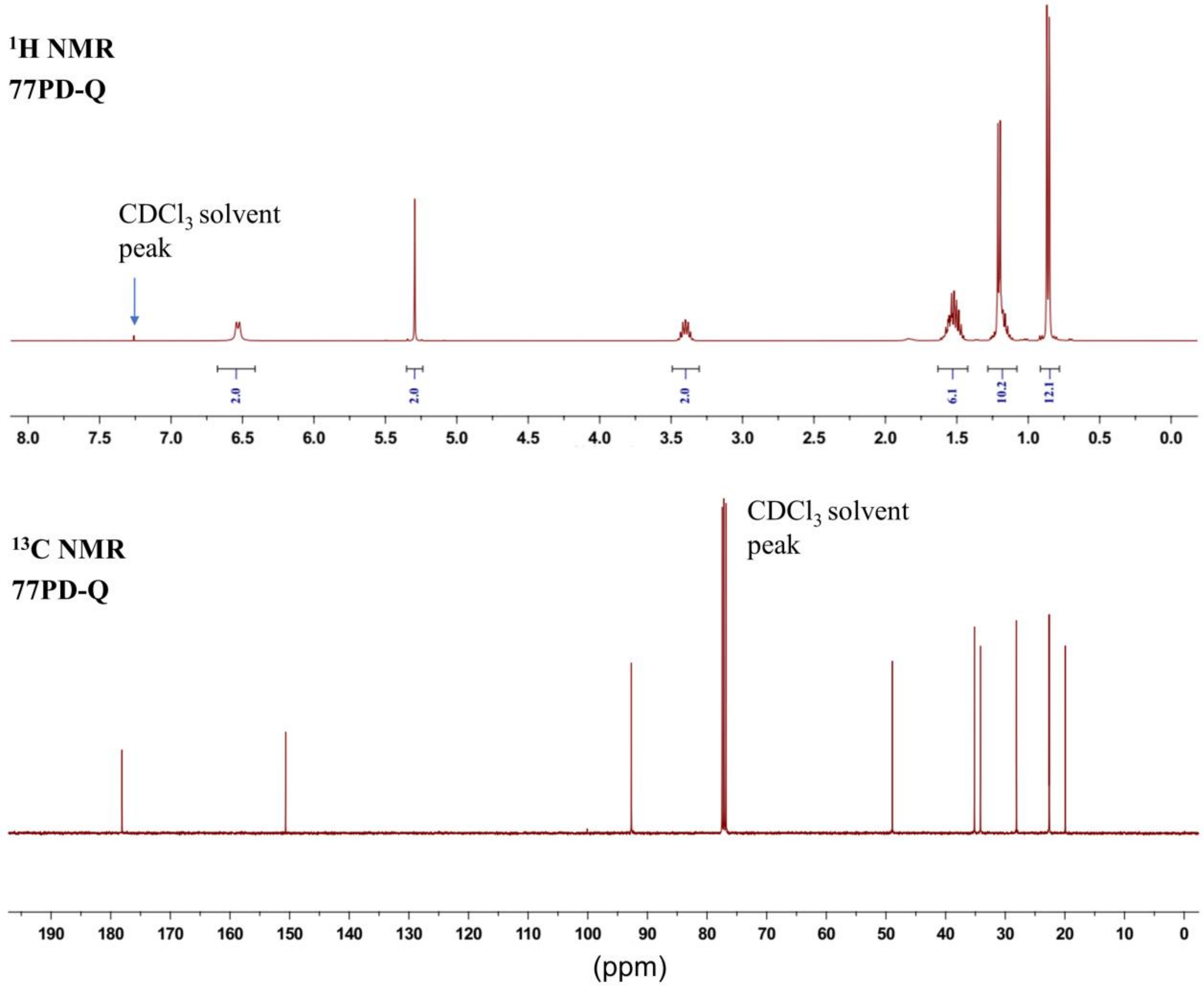

Figure S2. ^{1}H NMR (upper) and ^{13}C NMR spectra (lower) of 77PD-Q.

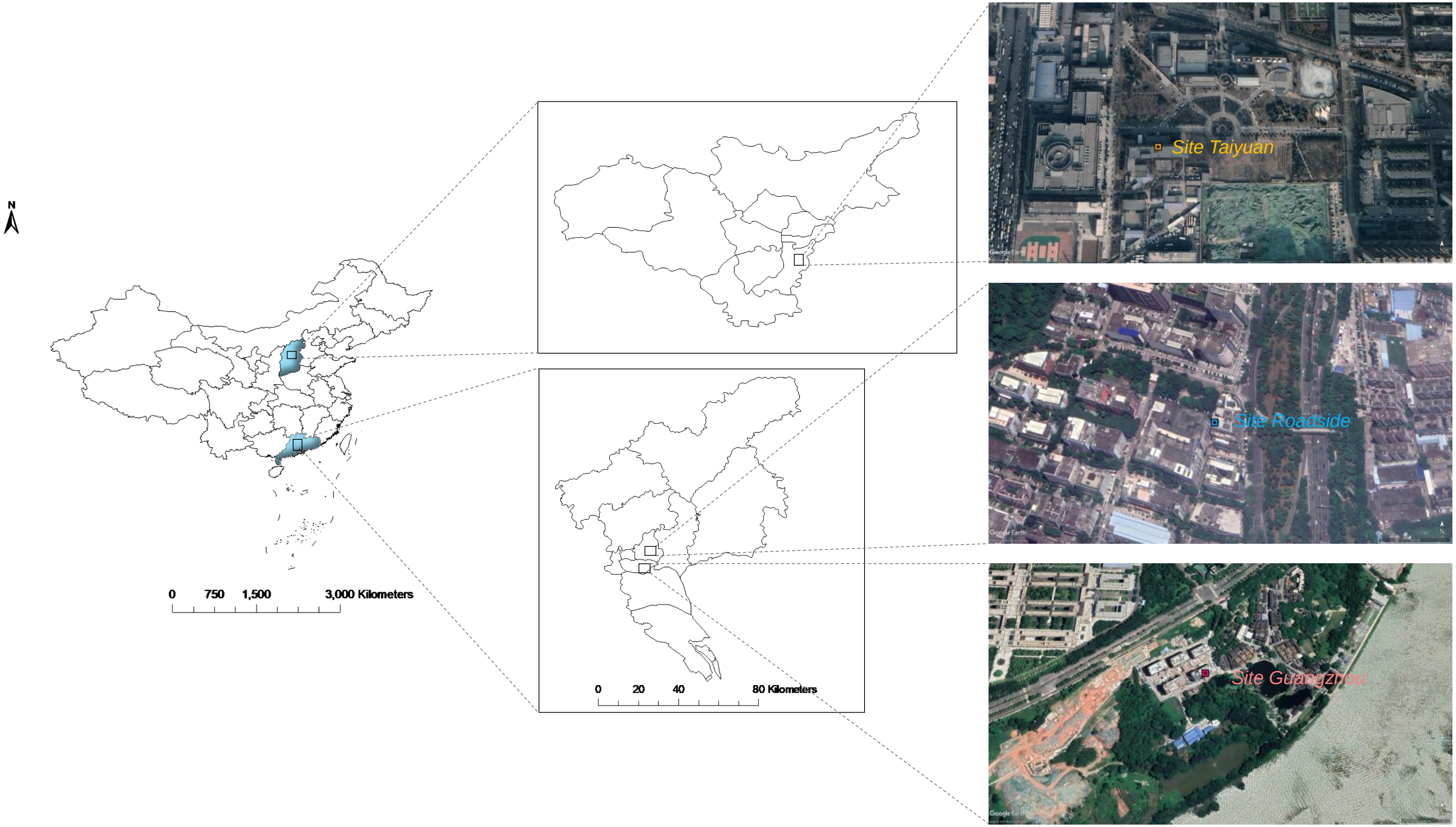

Figure S3. Specific geographical distribution of sampling site Guangzhou, Roadside and Taiyuan.

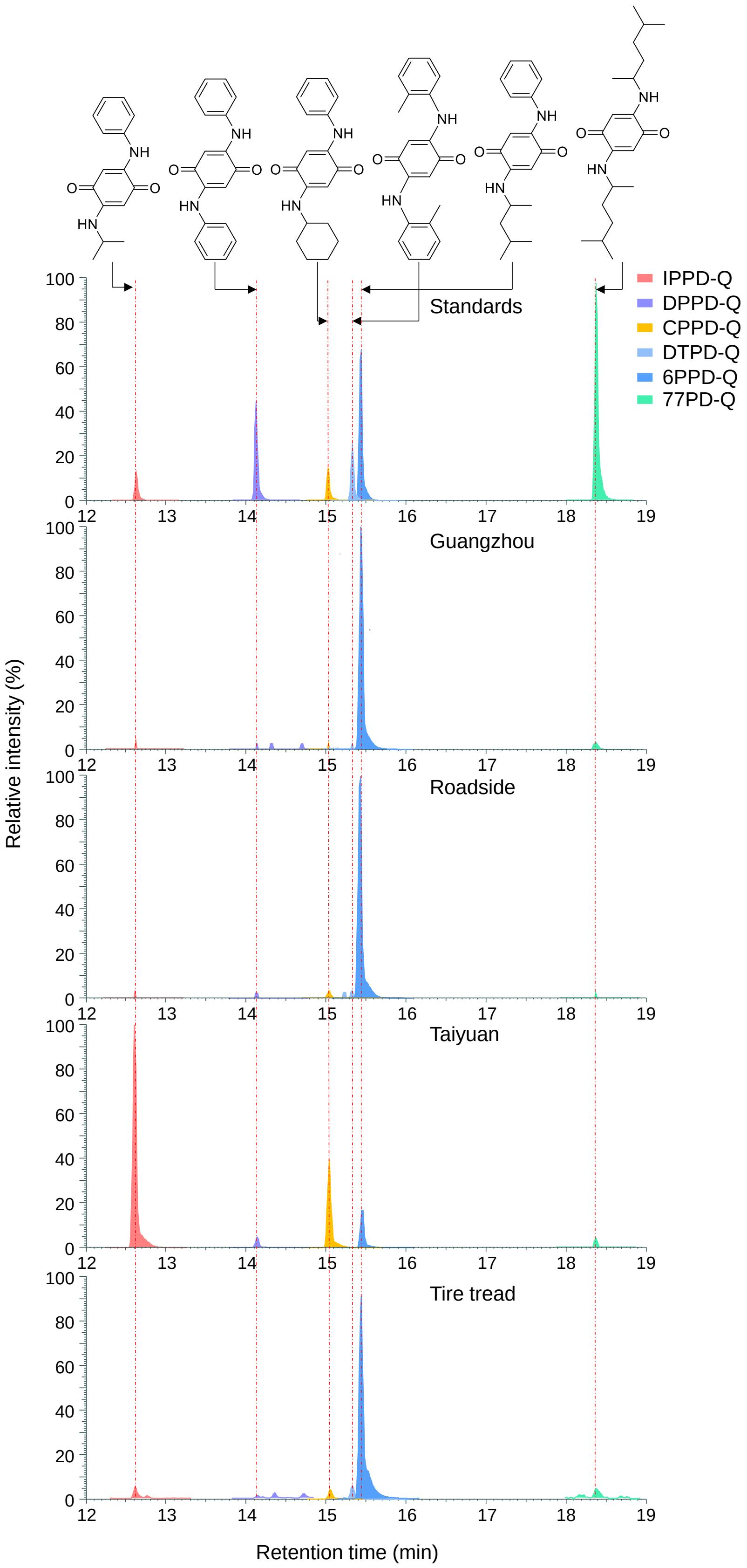

Figure S4. MRM chromatograms of the six analyzed PPD-Qs in standard solvent, $PM_{2.5}$ samples collected in site Guangzhou, roadside of Guangzhou, Taiyuan, and tire tread.

10PPDs_NCE10-20-40_New #3943-4011 RT: 12.61-12.79 AV: 7 NL: 3.02E6
F: FTMS + c ESI Full ms2 257.1291@hcd23.33 [50.0000

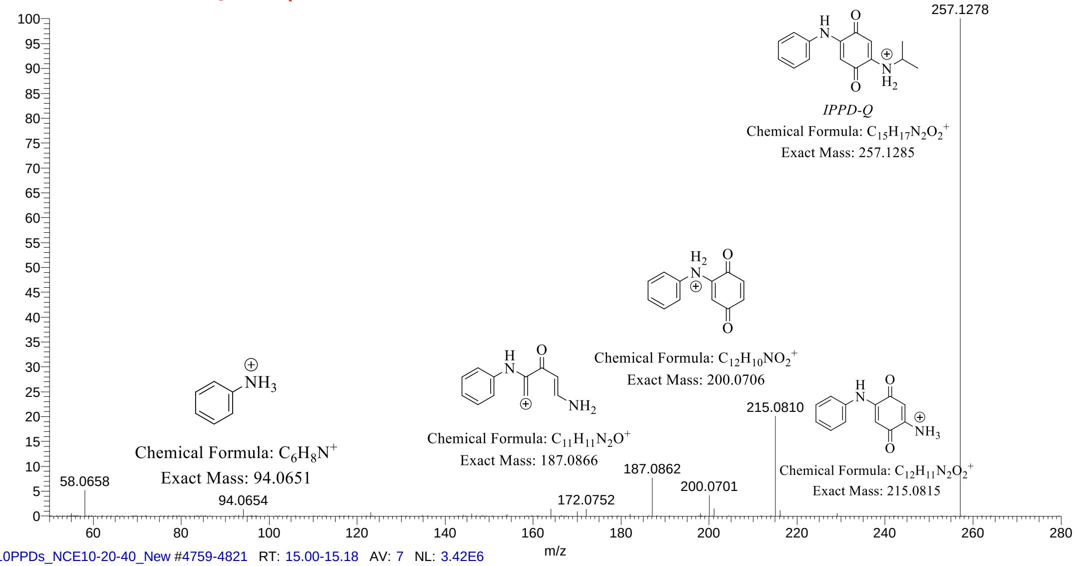

10PPDs_NCE10-20-40_New #4759-4821 RT: 15.00-15.18 AV: 7 NL: 3.42E6
F: FTMS + c ESI Full ms2 297.1604@hcd23.33 [50.0000

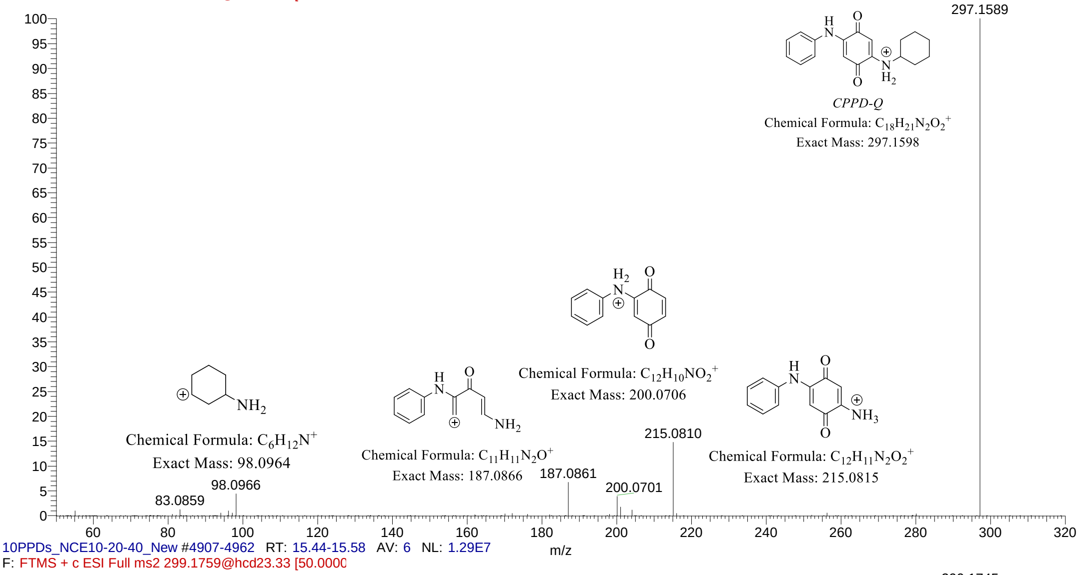

10PPDs_NCE10-20-40_New #4907-4962 RT: 15.44-15.58 AV: 6 NL: 1.29E7
F: FTMS + c ESI Full ms2 299.1759@hcd23.33 [50.0000

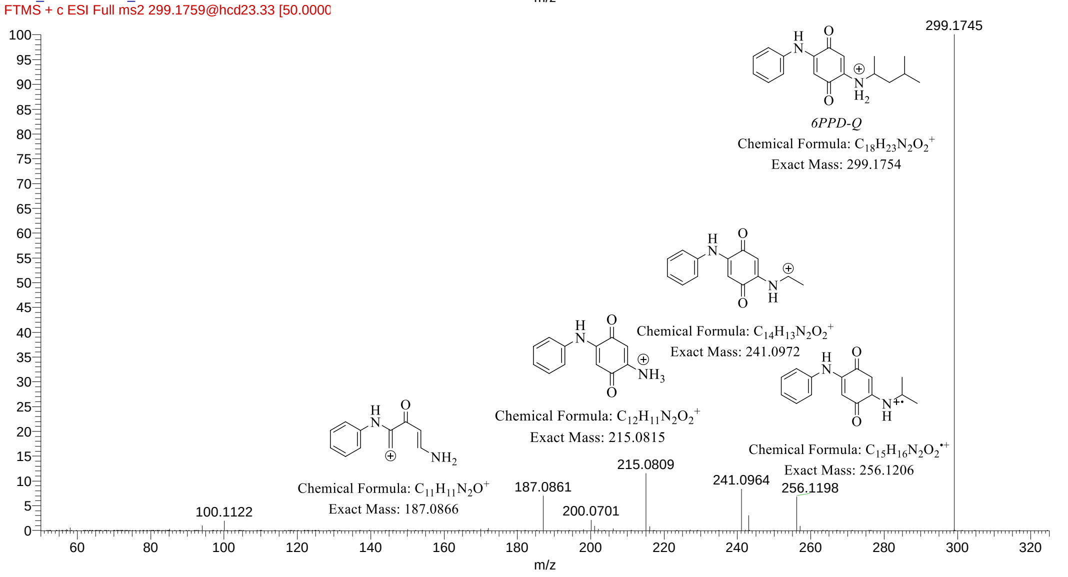

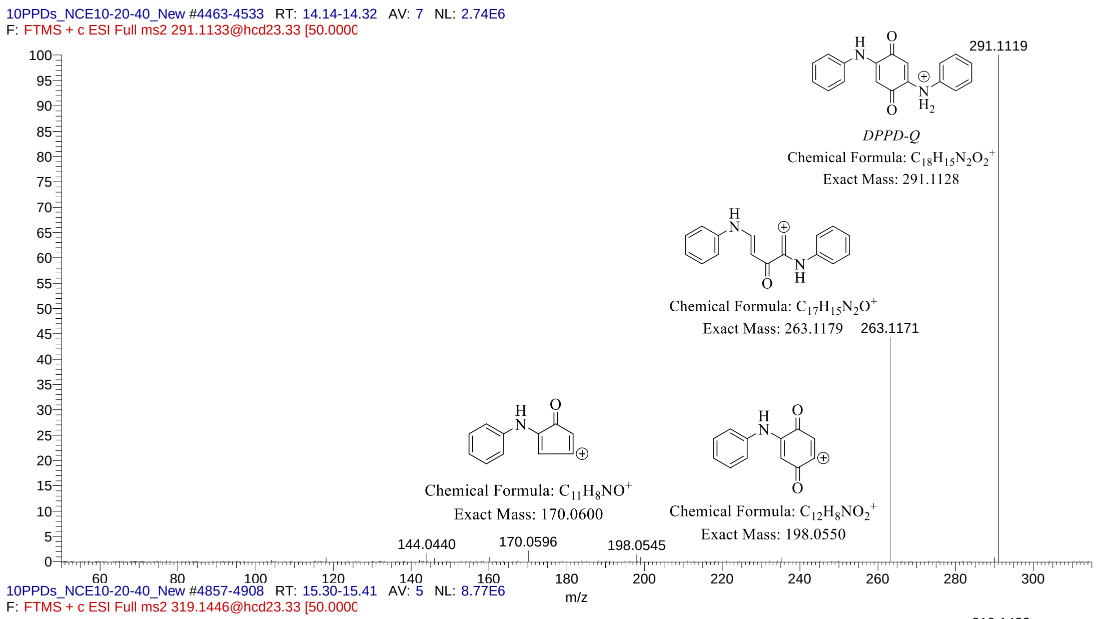

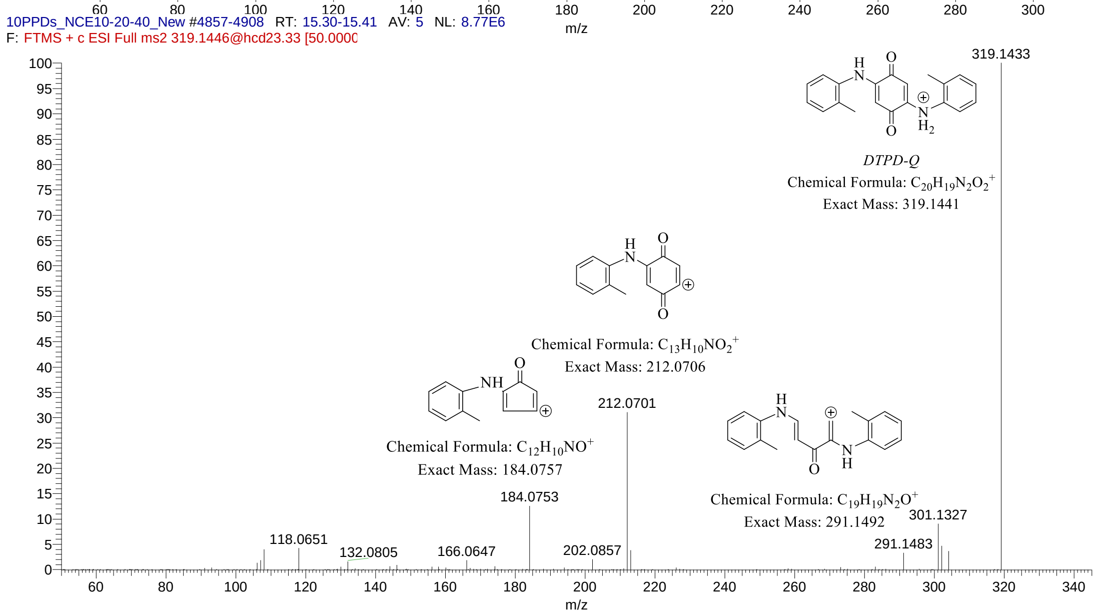

Figure S5. Specific fragmentation pathways for each PPD-Qs.

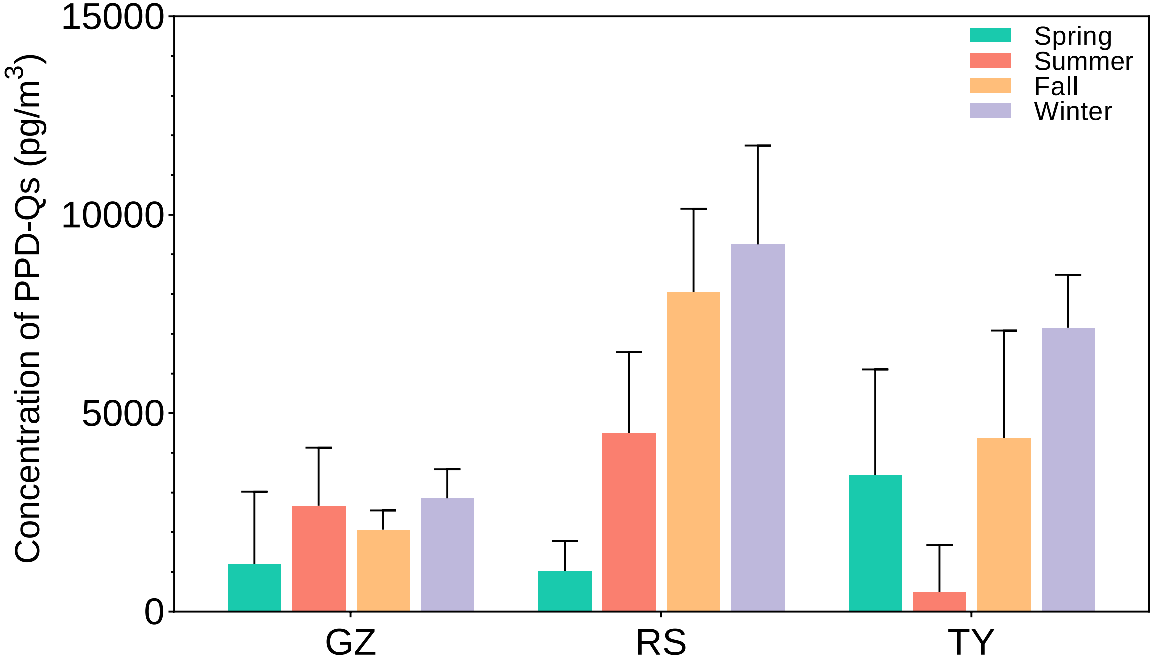

Figure S6. Seasonal concentration (mean ± S.D.) variation of PPD-Qs in Site Guangzhou (GZ), roadside of Guangzhou (RS) and Taiyuan (TY).

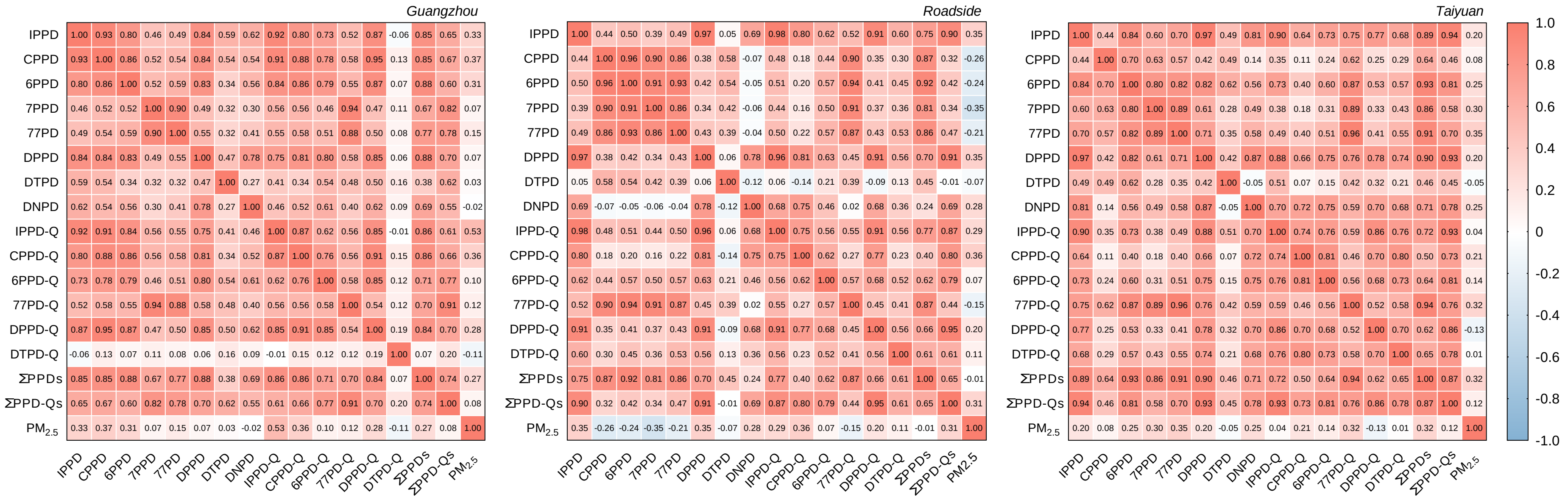

Figure S7. Spearman correlation coefficients among individual PPD and PPD-Qs in $PM_{2.5}$ from Guangzhou, Roadside, and Taiyuan. Site roadside is located in a near-street point that is also in City Guangzhou.

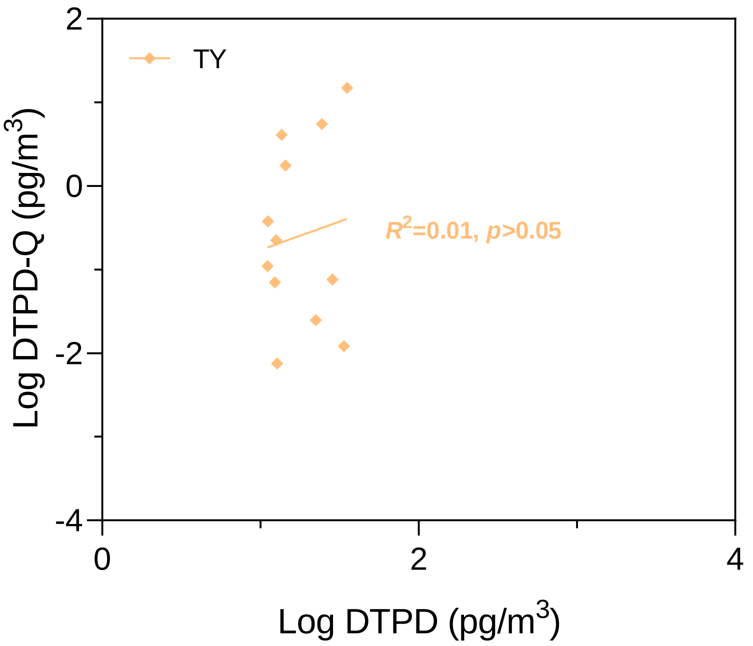

Figure S8. Linear regression between the concentration of DTPD-Q with DTPD. TY: site Taiyuan. Logarithm of data to base 10 (log_{10}). Site Guangzhou and Roadside were excluded due to their lower detection frequency of DTPD-Q (<50%).

References

1. USEPA, Risk Assessment Guidance for Superfund (RAGS) Volume I: Human Health Evaluation Manual (Part E, Supplemental Guidance for Dermal Risk Assessment) Interim. In 2009.
2. USEPA, Risk Assessment Guidance for Superfund Volume I: Human Health Evaluation Manual (Part F: Supplemental Guidance for Inhalation Risk Assessment). In 2019.
3. USEPA, *Exposure factors handbook: 2011 edition*, 2011.
4. Zhang, J.; Zhang, X.; Wu, L.; Wang, T.; Zhao, J.; Zhang, Y.; Men, Z.; Mao, H., Occurrence of benzothiazole and its derivates in tire wear, road dust, and roadside soil. *Chemosphere* **2018,** *201*, 310-317.
5. Ji, Y.; Wang, F.; Zhang, L.; Shan, C.; Bai, Z.; Sun, Z.; Liu, L.; Shen, B., A comprehensive assessment of human exposure to phthalates from environmental media and food in Tianjin, China. *J. Hazard. Mater.* **2014,** *279*, 133-40.
6. Cao, G.; Wang, W.; Zhang, J.; Wu, P.; Zhao, X.; Yang, Z.; Hu, D.; Cai, Z., New evidence of rubber-derived quinones in water, air, and soil. *Environ. Sci. Technol.* **2022**. DOI: 10.1021/acs.est.1c07376.
7. China National Environmental Monitoring Center, China National Urban Air Quality Real-time Publishing Platform. Available at: http://106.37.208.233:20035/emcpublish/ (*accessed* Aug 23, 2021)
8. National Meteorological Information Center, National Meteorological Information Center. Available at: http://data.cma.cn) (*accessed* Aug 23, 2021)
